# Effect of Pulsed Electric Fields (PEF) on Accumulation of Magnesium in *Lactobacillus rhamnosus* B 442 Cells

**DOI:** 10.1007/s00232-017-9986-6

**Published:** 2017-09-04

**Authors:** Małgorzata Góral, Urszula Pankiewicz

**Affiliations:** Department of Analysis and Food Quality Assessment, Faculty of Food Science and Biotechnology, University of Life Sciences, Skromna Street 8, 20-704 Lublin, Poland

**Keywords:** PEF, Magnesium, *Lactobacillus rhamnosus*, Bioaccumulation

## Abstract

The aim of this study was to determine the effect of pulsed electric fields (PEF) on accumulation of magnesium ions in *Lactobacillus rhamnosus* B 442 cells. Under optimized conditions, this is, on 15 min exposure of the 20 h grown culture to PEF of the 2.0 kV/cm and 20 µs pulse width at concentration 400 μg Mg^2+^/mL medium, accumulation of magnesium in the biomass reached maximum 4.28 mg/g d.m. Optimization of PEF parameters caused an increase of magnesium concentration in the cells by 220% in comparison to the control not treated with PEF. Bacterial cell biomass enriched with Mg^2+^ may be an alternative for pharmacological supplementation applied in deficiency of this cation.

## Introduction

Magnesium is the second intermolecular cation, besides calcium, which can be found in the largest amounts in the human body. The adult human body contains approximately 22–24 g of this element (Chaudhary et al. 2010; Akhtar et al. 2011). Magnesium plays a number of important functions: it is a cofactor of enzymatic reactions, participates in carbohydrates metabolism, and it is necessary for a proper functioning of many organs and tissues (Jahnen-Dechent and Ketteler 2012; Rylander 2014; Przybysz et al. 2016). The daily recommended intake of magnesium for males is 400–430 mg and for women is 300–320 mg, but it can be significantly increased for pregnant and nursing women, the elderly or persons under stress (Chaudhary et al. 2010; Jahnen-Dechent and Ketteler 2012).

The studies proved that excessive consumption of processed food causes a magnesium deficiency. About 50–80% of U.S. population shows deficit of this element. Overconsumption of sugar, alcohol, and caffeine as well as taking diuretics is responsible for magnesium loss (Chaudhary et al. 2010). Early symptoms of magnesium deficiency include, among others, fatigue, lack of appetite, irritation, insomnia, and muscle cramp. Some people may also be affected by anxiety, impairment of learning ability, and memory problems (Slutsky et al. 2010). Low magnesium levels increase risk of heart diseases, high blood pressure, and lithiasis. It can also be a reason of the higher susceptibility to depression (Eby and Eby 2010; Derom et al. 2013). The prolonged magnesium deficiency leads to numbness, formication, and long-lasting muscle cramps or even hallucinations (Laires et al. 2004).

Supplementation of diet with magnesium ions may guarantee a proper functioning of the organism. A perfect source of minerals should be highly available for human organism, safe in application and must have the desirable physical and chemical properties (Ziarno et al. 2009). Rich sources of magnesium are wheat, brown rice, sweet corn, barley, soy, sweet potatoes, broccoli, tomatoes, and papaya (Marles 2017).

Metal ions are better tolerated and assimilated in the form of metalloproteins which can be found in microorganisms (Knoop et al. 2005; De Nicola and Walker 2009; Mrvčić et al. 2012). Magnesium ions occur naturally in the cells of lactic acid bacteria (LAB). They are essential for stimulation of enzyme production (Givry and Duchiron 2008). Probiotic strains of *Lactobacillus* (GRAS, generally recognized as safe), are widely used in the food industry, especially in the production of fermented foods. They are included to a group of Gram-positive bacteria as they contain significant amount of teichoic acid and peptidoglycan taking a part in the biosorption process (Monachese et al. 2012; Kinoshita et al. 2013).

Metal ions are bounded by the surface of bacteria consisting mainly of polysaccharides and take part in processes of ion exchange, complexion, chelation, and microprecipitation. Extent of these processes depend on a number of functional groups on the surface of cells, type of metal ions and their concentration, as well as surface charge. After saturation of available binding sites on the cell wall, metal ions are accumulated in the protoplast. As soon as some of the binding sites become free, they are filled again with external metal ions until the state of equilibrium is achieved (Monachese et al. 2012; Mrvčić et al. 2012).

Pulsed electric field technology (PEF) can be applied for enrichment of bacteria in ions. In a cell subjected to PEF, induced trans-membrane tension facilitates the formation of pores in the membrane and leads to an increase in its permeability (Dellarosa et al. 2016; Traffano-Schiffo et al. 2016). The effects of this process depend on voltage applied, e.g., in the range of voltage from 0.5 to 1 V arising of new pores with diameter of several nanometers is observed. Permeability of cell membrane increases secretion of compounds inside a cell or accumulation of ions in a protoplast. At the optimized parameters of the process electroporation is reversible (Pankiewicz and Jamroz 2010; Pankiewicz et al. 2014; Barba et al. 2015). Electric field strength of 0.7–3.0 kV/cm may cause irreversible membrane breakage and destruction of cellular structures. Cell loses an ability to maintain its natural physiological processes (Gehl 2003). Phenomenon of electroporation was used, among others, by Pankiewicz et al. (2015) for enrichment of *Saccharomyces cerevisiae* cells in metal ions. The aim of the study was to determine the effect of pulsed electric field (PEF) on the accumulation of magnesium ions in *Lactobacillus rhamnosus* B 442 cells.

## Materials and Methods

A strain of *Lactobacillus rhamnosus* B 442 from the collection of Department of Biotechnology, Human Nutrition and Science of Food Commodities, University of Life Sciences and Biotechnology in Lublin, Poland was used in the experiment. For the preparation of inoculum and culture medium, the following components were used: sterile (sterilization at 121 °C and 0.5 atm for 20 min), MRS broth (BTL, Łódź, Poland) 58.937 g/L, Tween 80 (Biochemica, ICI, USA) 1 mL/L, agar (DIFCO, Detroit, MI, USA) 15 g/L, NaCl (POCH, Gliwice, Poland) 80 g/L, glycerol (TechlandLab, Tarnobrzeg, Poland), HNO_3_ 65% (Merck, Darmstadt, Germany), and MgCl_2_ × 6 H_2_O (POCH, Gliwice, Poland) in the fixed concentrations.

## Biomass Cultivation

Bacteria were passaged three times in MRS broth (components in 1 L: yeast extract 4 g, ammonium citrate 2 g, beef extract 8 g, K peptone 10 g, glucose 2 g, dipotassium phosphate 2 g, sodium acetate 5 g, magnesium sulfate ×7 H_2_O 0.2 g, magnesium sulfate ×4 H_2_O 0.05 g) with Tween 80 and incubated at 37 °C for 24 h. Then inoculum was prepared by transferring 3 mL of bacteria to 57 mL of sterile medium in 500-mL Erlenmeyer flasks. Flasks were incubated at 37 °C for 48 h. The obtained inoculum (5 mL) was transferred into 85 mL of sterile medium placed in Erlenmeyer flasks. An 10-mL aliquots of magnesium chloride at fixed concentration was added to each flask (except of the control sample K1). Then the culture was incubated at 37 °C for 24 h.

### Optimization of Magnesium Concentration in Cells and PEF Parameters

Optimization of magnesium concentration in the medium was performed by culturing of *L. rhamnosus* B 442 at different concentrations of magnesium (μg/mL medium): 10, 40, 100, 200, 400, 600, 800, 1000. After 16 h of incubation, cultures were treated with PEF for 10 min at pulse width of 10 μs, electric field strength of 2.0 kV/cm, using a laboratory electroporator (BTX Harward Apparatus, model ECM 830). Then the cultures were incubated for 19 h. Simultaneously, the control cultures were conducted, respectively, K1—without magnesium added to the medium and without PEF treatment and K2—with magnesium added to the medium in the concentrations mentioned above and without PEF. The PEF treatment chamber consisted of four parallel plexiglas plates which had stainless steel electrodes of an area equal to 4 cm^2^, facing each other with a gap of 5 mm. The culture was agitated in a chamber during PEF treatment with a magnetic stirrer.

Optimization of PEF parameters was carried out in a few stages. In the first stage, the optimal electric field strength was set by subjecting of the cultures after 16 h of incubation to electroporation at the range of electric field strength 0.1–3.5 kV/cm, exposition time of 10 min, pulse width of 10 μs, and frequency of 1 Hz. Microorganisms were treated with PEF at the optimal concentration of magnesium (400 μg/mL medium) which was set earlier (section: optimization of magnesium concentration in cells and PEF parameters). In the next stage, the time of electroporation was optimized in the range 5–25 min, at optimal electric field strength (2.0 kV/cm) fixed earlier. In the following step, pulse width was optimized in the range 50–150 μs at optimal PEF parameters: electric field strength 2.0 kV/cm, electroporation time 15 min, and frequency 1 Hz. In the last stage optimization of the time of incubation after which yeast cells were treated with PEF was performed. Cells were electroporated after 8, 12, 16, 20, and 24 h of culturing, and one of the samples was subjected to a multiple treatment with PEF after 8, 12, 16, 20, and 24 h of culturing. In both cases, the optimal PEF parameters were applied: electric field strength 2.0 kV/cm, electroporation time 15 min, pulse width 20 μs, and frequency 1 Hz.

### Determination of Magnesium Concentration in Biomass

In order to determine concentration of magnesium in cells, biomass was centrifuged (15 min, 3000 rpm, 1467 g), supernatant was discarded, and cells were rinsed three times with deionized water. Then biomass was lyophilized in a Labconco freeze dryer (model 64,132, Kansas City, MO, USA) and mineralized in a MARS microwave oven (CEM Corporation, USA). Samples were prepared as follows: about 0.1 g of lyophilizate was transferred to a tube and 3 ml of HNO_3_ was added. Then the samples were mineralized at 200 °C for 20 min. The obtained solutions were cooled down, transferred to 25 mL measuring flasks and topped up with deionised water. Concentration of magnesium ions in *L. rhamnosus* B 442 cells was determined using an electrothermal atomic absorption spectrophotometer (ET-AAS, VARIAN AA 280 FS) according to BS EN 15505: 2008.

### Determination of Cell Vitality

Total number of microorganisms was determined by plate dilution method according to the American Public Health Association (1993). Colonies of microorganisms were diluted 8 and 9 times with 0.8% sterile NaCl. Then 1 cm^3^ of each was taken and placed in Petri dishes in two replicates. The diluted bacteria were flooded with sterile liquid agar. Cultures were incubated for 48 h at 37 °C. The Petri dishes with two consecutive dilutions on which grew from 25 to 250 colonies were selected for reading. The total number of microorganisms (L) in 1 cm^3^ of the sample was calculated according to the formula (1):

1 $$L = \frac{C}{{\left( {N_{1} + 0.1N_{2} } \right)}} \times d,$$

where *C* sum of colonies on all dishes selected for counting, *N*_1_ number of dishes from the first calculated dilution, *N*_2_ number of dishes from second calculated dilution, *d* dilution ratio corresponding to the first (lowest) dilution.

### Determination of Biomass

Biomass was determined spectrophotometrically (Spekol 11, Carl Zeiss, Jena, Germany). A sample of the culturing medium (2 mL) was centrifuged, supernatant was discarded, cells were rinsed three times with deionized water and brought to the original volume of 2 mL. Turbidimetric measurements were run against pure water at *λ* = 600 nm, in 2 mm measurement cell. Amount of biomass was calculated using equation for the standard curve *c* (mg/mL) = (Ap + 0.1053):1.1511, where Ap apparent absorbance (Pankiewicz and Jamroz 2010).

### Statistical Analysis

Significant differences between particular groups were found using the Student *t* test applied to compare independent samples in pairs, and variance analysis (ANOVA) was used for more than two groups. Detailed analysis was based on Tukey’s confidence intervals. All statistical tests were carried out at significance level of *α* = 0.05. Statistical processing of results was performed using R program version 3.1.2.

## Results and Discussion

Magnesium, in the form of MgCl_2_, is recommended for supplementation of media used for lactic acid bacteria (LAB) culturing and it has no negative influence on bacterial cells. Appropriate amounts of this element are essential for synthesis of DNA and proteins (Watanabe et al. 2012; Zhang et al. 2014). Ability of microorganisms to grow in the presence of high metal concentrations may result from their mechanisms of resistance to oxidative stress: change of redox state of the metal ions, precipitation of metals and extracellular complexation (Klaus-Joerger et al. 2001; Valko et al. 2005). Magnesium plays an important role in the synthesis of proteins and nucleic acids and cell cycle. Cations of this metal together with phospholipids constitute structural complexes of cell membranes affecting their stability and permeability (Roman et al. 2009).

**Fig. 1:**
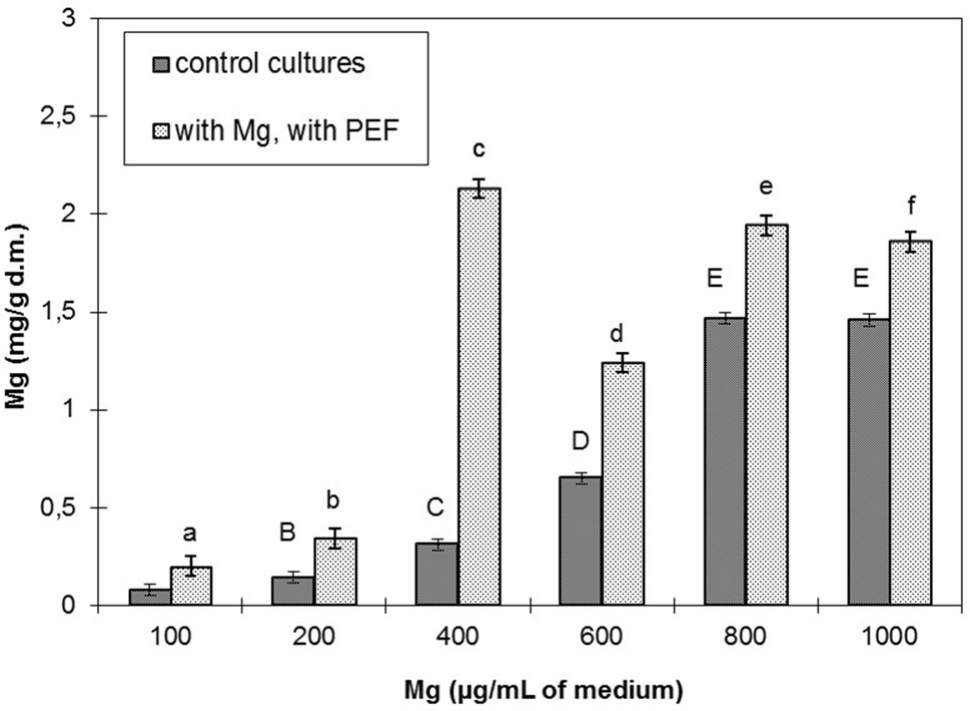
Effect of the magnesium concentration in the medium upon magnesium accumulation in *L. rhamnosus* B 442 (electric field strength 2.0 kV/cm, pulse width 10 μs, field frequency 1 Hz, time of exposure to PEF 10 min after 16 h of cultivation). Means with the *same small* or *large letters* are not highly significantly different (*P* < 0.05; *n* = 6). Control cultures without PEF and supplemented with magnesium ions

The studies revealed that increasing concentration of magnesium in the range 100–1000 μg/mL medium led to its higher accumulation in cells from the cultures not treated with PEF (K2). In the medium of 1000 μg Mg/mL, a slight decline in accumulation of this element was observed in the cultures treated with PEF. The highest accumulation (2.13 mg Mg/g d.m.) was reached in the culture *L. rhamnosus* B 442 electroporated at a concentration of 400 μg Mg/mL medium (Fig. 1), and it was sixfold higher than in the control culture K2. The relationship between biomass increment and magnesium concentration was statistically significant both in the control K2 and the electroporated culture. Biomass was in the range from 0.22 to 0.31 g/g d.m.

In the electroporated cultures, in contradiction to the control cultures, the increase of cell vitality together with increasing Mg concentration was observed (Table 1). The nonlinear dependence of this parameter upon concentration of magnesium in the medium was significant both in the cultures treated (third degree polynomial regression, *R* ^2^ = 0.9876) and not treated with PEF (exponential regression, *R* ^2^ = 0.8572). Cell vitality (at concentration of magnesium 400 μg/mL and with PEF treatment) dropped by 1.39 × 10^8^ cfu/mL as compared to the culture K1 (without supplementation with magnesium and PEF treatment). Loghavi et al. (2008), who analyzed an effect of moderate electric field frequency on growth kinetics and metabolic activity of *Lactobacillus acidophilus*, reported that PEF frequency of 45 and 90 Hz had no significant influence on cell growth. Gniewosz et al. (2014) showed that increased amount of magnesium in the medium had considerable influence on total bacterial numbers determined for the selected strains. Medium was enriched in magnesium at a concentration of 1.25 g/L and the obtained results of vitality determination were in the range from 1.15 × 10^9^ to 2.52 × 10^9^ cfu/mL. These values were higher than those received in this study. Addition of magnesium did not cause a decrease of cell vitality in comparison to the control cultures, unlike the vitality of the bacteria evaluated by the authors. Cao et al. (2009) investigated influence of Mg^2+^ on the growth and activity of sulfate reducing bacteria. They noted that level of magnesium accumulation was 0.59 mg/L at supplementation dose of 1 g MgSO_4_·7H_2_O/L medium. Lew et al. (2013) reported that dosage of 1.09 mg MgSO_4_/mL was optimal for the growth of *Lactobacillus rhamnosus* FTDC 8313. The studies of Roman et al. (2009) revealed that addition of magnesium acetate to a culturing medium caused the increase of Mg accumulation in the *L. brevis* and *L. plantarum* ATCC biomass. They noted higher magnesium accumulation than those shown in the part of this study not including the optimization of PEF parameters (2.65–5.82 mg Mg g^−1^s.s for *L. brevis* and from 1.73 to 3.28 mg Mg g^−1^s.s. for *L. plantarum* ATCC).

**Table 1:** Influence of PEF treatment and magnesium concentration in medium on vitality and biomass of *Lactobacillus rhamnosus* B 442

Concentration of magnesium (μg/mL)	Vitality (cfu/mL)		Biomass (g/g d.m.)	
	Without PEF	With PEF (2,0 kV/cm, 10 μs, 10 min)	Without PEF	With PEF (2,0 kV/cm, 10 μs, 10 min)
K1	1.43 × 10^8^	–	0.286	–
10	6.81 × 10^7^	2.82 × 10^6^	0.276	0.227
40	3.71 × 10^7^	9.45 × 10^6^	0.239	0.244
100	8.41 × 10^7^	1.82 × 10^6^	0.279	0.269
200	9.80 × 10^7^	2.15 × 10^6^	0.267	0.25
400	3.67 × 10^7^	3.55 × 10^6^	0.281	0.24
600	1.19 × 10^7^	6.36 × 10^5^	0.315	0.23
800	6.09 × 10^6^	2.18 × 10^7^	0.259	0.258
1000	4.91 × 10^6^	8.09 × 10^7^	0.271	0.244

*Control culture K1* without PEF treatment and addition of magnesium ions

**Fig. 2:**
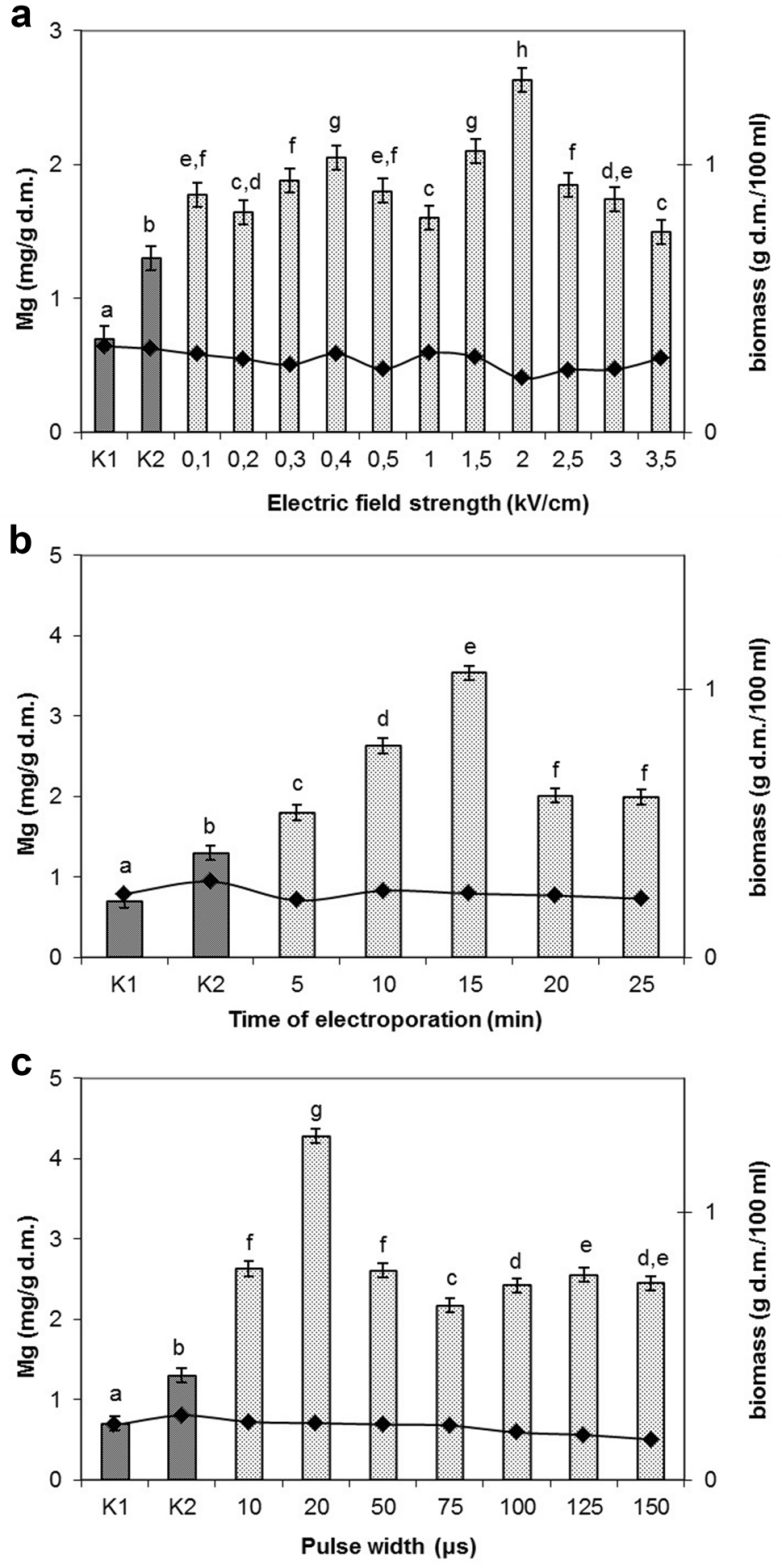
Accumulation of magnesium (magnesium concentration in the culture medium 400 μg/mL) in *L. rhamnosus* B 442 cells depending on: **a** electric field strength (electric field strength between 0.1 and 3.5 kV/cm, pulse width 10 μs, field frequency 1 Hz, time of exposure to PEF 10 min after 16 h of cultivation). **b** Time of exposure to PEF (the time of exposure to PEF between 5 and 25 min after 16 h of cultivation, pulse width 10 μs, electric field strength 2.0 kV/cm, field frequency 1 Hz). **c** Pulse width (pulse width between 10 and 150 μs, electric field strength 2.0 kV/cm, field frequency 1 Hz, the time of exposure to PEF 15 min after 16 h of cultivation). Means with the *same letters* are not highly significantly different (*P* < 0.05). *Control cultures K1* without magnesium and without PEF treatment; *K2* with magnesium (400 μg/ml) and without PEF

Magnesium concentration of 400 μg/mL medium was assumed as the optimal for bioaccumulation of this element in *Lactobacillus rhamnosus* B 442 cells and applied in the further experiments. The results demonstrated a positive effect of pulsed electric field on accumulation of magnesium ions in *L. rhamnosus* B 442 cells. The highest accumulation, which was over twofold higher than in the control K2, was reached at electric field strength of 2.0 kV/cm (Fig. 2a). When a value of this parameter was higher than 2.0 kV/cm a drop of Mg accumulation was noted. At 0.1, 0.3, 0.5, and 3.0 kV/cm there were no significant differences in vitality in comparison to the control cultures K1 and K2 (Table 2). A high drop of vitality was noted at 0.4 and 3.5 kV/cm by, respectively, 2.72 × 10^8^ and 2.73 × 10^8^ cfu/mL as compared to K2. The statistically significant changes in biomass were observed at electric field strength of 0.2, 0.3, 0.5 kV/cm and in the range from 2.0 to 3.5 kV/cm. Biomass content was in the range 0.2–0.3 g/g d.m. Pankiewicz and Jamroz (2010) obtained the highest accumulation of magnesium in *S. cerevisiae* cells treated with PEF at twice the field strength than the one used in this study (4.0 kV/cm). At lower values of electric field strength (0.1–1.0 kV/cm) higher accumulation was not observed. However, the authors noted an increase in Mg^2+^ accumulation in the mentioned interval (0.1–1.0 kV/cm), as compared to the control K2 but at the values over 2.0 kV/cm accumulation was enhanced reaching maximum at 4.0 kV/cm. The research conducted by Najim and Aryana (2013) showed that application of pulsed electric field of the following parameters: electric field strength of 1.0 kV/cm, pulse width of 3 μs, and pulse period of 0.5 s significantly affected growth of *L. acidophilus* LA-K and *L. delbrueckii* ssp*. bulgaricus* LB-12. The authors concluded that PEF can be recommended for pretreating cultures to enhance desirable attributes of LAB used for production of probiotic foods. The bacterial vitality values obtained in this study at 1.0 kV/cm were also higher in comparison with the control K1. Seratlic et al. (2013) showed that treatment of *Lactobacillus plantarum* 564 cells with pulsed electric fields at field strength less than 13.6 kV/cm did not reduce the vitality of bacteria. The use of suitable parameters of culturing after PEF application allows the pores to be sealed and the cell returned to physiological state. The authors of this paper did not carry out research at such a high field strength.

**Table 2:** Effect of electric field strength, time of exposure to PEF, pulse width, and time of cultivation on vitality of *Lactobacillus rhamnosus* B 442

Vitality at low electric field strength (cfu/mL)		Vitality at high electric field strength (cfu/mL)		Vitality at various time of electroporation (cfu/mL)		Vitality at various pulse width (cfu/mL)		Vitality at various cultivation time (cfu/ml)	
K1	2.84 × 10^8^ b	K1	6.05 × 10^8^ e	K1	1.23 × 10^11^ g	K1	2.64 × 10^9^ e	K1	3.15 × 10^9^ c
K2	2.80 × 10^8^ b	K2	9.37 × 10^8^ g	K2	9.37 × 10^8^ a	K2	9.37 × 10^8^ a	K2	9.37 × 10^8^ a
0.1	2.77 × 10^8^ b	1.5	6.79 × 10^8^ f	5	3.05 × 10^9^ b	10	1.59 × 10^9^ a	8	1.16 × 10^10^ e
0.2	6.91 × 10^8^ c	2.0	1.56 × 10^8^ c	10	4.98 × 10^9^ c	20	2.65 × 10^9^ b	12	2.82 × 10^9^ b
0.3	2.56 × 10^8^ b	2.5	1.72 × 10^8^ d	15	3.81 × 10^10^ f	50	3.51 × 10^9^ bc	16	8.59 × 10^9^ d
0.4	8.25 × 10^7^ a	3.0	1.01 × 10^8^ b	20	1.82 × 10^10^ d	75	3.61 × 10^9^ bc	20	1.25 × 10^10^ f
0.5	2.71 × 10^8^ b	3.5	6.91 × 10^6^ a	25	3.31 × 10^10^ e	100	1,13 × 10^10^ d	24	3.15 × 10^9^ c
1.0	2.31 × 10^9^ d					125	1.45 × 10^9^ a		
						150	3.97 × 10^9^ c		

*Control cultures K1* without magnesium and without PEF treatment; *K2* with magnesium (400 μg/ml) and without PEF treatment. Means with the same letters within a column do not differ significantly (*P* < 0.05)

The studies on time of exposure to PEF were conducted in the range of 5–25 min (Fig. 2b). Initially, extending electroporation time to 15 min caused significant increase of magnesium accumulation in the cells. It was the highest after 15 min of treatment when magnesium content in the cells amounted to 3.54 mg/g d.m. (over twofold higher than in the culture K2). Further extending of exposure time to 20 and 25 min reduced Mg accumulation in the cells. Time of exposure to PEF affected significantly (nonlinear dependence) biomass production (third degree polynomial regression, *R* ^2^ = 0.9385) and cell vitality (power regression, *R* ^2^ = 0.7776) (Table 2). The cultures electroporated for 5, 15, and 25 min differed significantly from the control culture K2. Pankiewicz and Jamroz (2011) reported that accumulation of zinc in *S. cerevisiae* cells was maximal after 15 min exposure to PEF. Despite the same optimal time of exposure to PEF was applied, in this study significantly lower ion accumulation was received. This may be due to the use of bacteria with different cell structure. By contrast, Pankiewicz et al. (2017) showed that the electroporation time had a significant effect on the simultaneous accumulation of selenium and zinc in *S. cerevisiae* cells. The authors found that the optimum time for bioaccumulation of both ions was 10 min. Application of this time of exposure to PEF caused 115% (for Se^4+^) and 150% (Zn^2+^) higher metal concentrations in yeast cells compared to the non-electroporated control sample. Prolongation of electroporation resulted in a decrease in bioaccumulation of ions.

Pulse width was optimized in the subsequent step of the studies (Fig. 2c). The highest concentration of magnesium ions, even threefold higher than in the culture K2, was noted at pulse width of 20 μs. Accumulation of magnesium in the cells electroporated at 10 and 50 μs as well as at 100 and 150 μs did not differ significantly. Pulse width did not affect biomass production, but there were statistically significant differences in vitality of cells between the cultures treated at 20, 50, 75, 100, and 150 μs and the control culture K2 (Table 2). Pankiewicz and Jamroz (2011) obtained the highest accumulation of zinc in *S. cerevisiae* cells (15 mg/g d.m.) when pulse width of 10 μs was applied. Fox et al. (2008) showed that a pulse width of 2 μs–300 μs did not cause lethal effect in *L. plantarum* cells. No lethality was observed in this study after applying pulse width in range from 10 to 150 μs. There was even an increase in the vitality of microorganisms at higher pulse width values compared to the control K2.

**Fig. 3:**
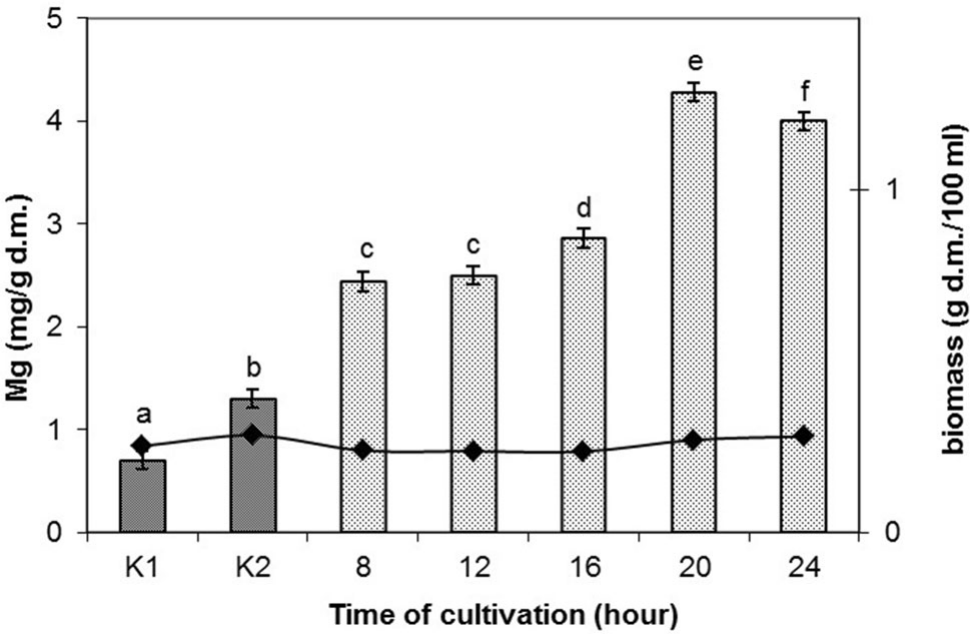
Effect of time for which cultures were treated with PEF upon magnesium concentration in *L. rhamnosus* B 442 (pulse width 20 μs, electric field strength 2.0 kV/cm, field frequency 1 Hz, time of exposure to PEF 15 min after 8, 12, 16, 20, or 24 h of cultivation; magnesium concentration in the culture medium 400 μg/ml). Means with the *same letters* are not highly significantly different (*P* < 0.05; *n* = 7). *Control cultures K1* without magnesium and without PEF treatment; *K2* with magnesium (400 μg/ml) and without PEF

The time after which bacterial cells were treated with PEF had also a significant influence on accumulation of magnesium ions. There were no significant differences in accumulation of this element in the cells after 8 and 12 h of culturing. Maximum value (4.28 mg Mg^2+^/g d.m.) was noted when cells were electroporated after culturing for 20 h and it was 2.9-fold higher in comparison to the culture K2 (Fig. 3). The time after which cultures were electroporated had no significant influence on content of bacterial biomass in relation to the control cultures K1 and K2. There were no significant differences in biomass production between cultures after 8, 12, 16, and 24 h of culturing. Vitality of bacteria reached 2.89 × 10^9^ to 1.25 × 10^10^ cfu/mL (Table 2). Significant differences in vitality were noted for the cultures treated with PEF after 8, 12, 16, and 24 h. Magnesium accumulation in the cells after a multiple electroporation amounted to 2.783 mg/g d.m. The studies showed that a multiple exposition of bacterial culture to PEF did not increase the accumulation of magnesium in cells, but caused even a 1.5-fold drop of this value as compared to maximum accumulation noted for a single treatment with PEF. Duszkiewicz-Reinhard et al. (2002) reported threefold increase of magnesium accumulation in the 24-h culture of *S. cerevisiae* (9 mg Mg/g d.m.) in comparison to the culture not supplemented with this element. Seratlić et al. (2013) who studied survivability of *Lactobacillus plantarum* 564 population treated with PEF showed that bacteria subjected to a multiple electroporation demonstrated higher resistance to further PEF application. The researchers applied electroporation after 3 h from completion of 3-day incubation of the culture at electric field strength of 22.9 and 31.6 kV/cm, pulse width of 5 μs and period between pulses of 10 ms.

## Conclusions

The results demonstrated that the application of PEF increased the accumulation of Mg^2+^ in *Lactobacillus rhamnosus* B 442 cells. Bioaccumulation of this metal depended on PEF parameters and Mg concentration in the medium. Electroporation of bacteria at the following parameters: Mg concentration 400 μg/mL medium, electric field strength 2.0 kV/cm, electroporation time 15 min, pulse width 20 μs, and time of culturing 20 h caused the increase of bioaccumulation by 2.89 mg/g d.m. (220%) in comparison to the control sample not treated with PEF (K2).

Bacterial biomass at the optimized PEF parameters changed in the range from 0.201 to 0.281 g/g. Electroporation had a significant impact on cell vitality. There was an increase in microbial survival at high magnesium concentrations (800 and 1000 mg/mL). However no linear relationship between bacterial vitality and change of PEF parameters was observed. Its highest value (3.81–10^10^ cfu/mL) was noted after electroporation of the cultures for 15 min. Medium supplementation with magnesium stimulated activity of microorganisms and did not show any bactericidal effect.
